# Efficacy and Safety of Botulinum Toxin Type A on Persistent Myofascial Pain: A Randomized Clinical Trial

**DOI:** 10.3390/toxins12060395

**Published:** 2020-06-15

**Authors:** Giancarlo De la Torre Canales, Natalia Alvarez-Pinzon, Victor Ricardo Manuel Muñoz-Lora, Leonardo Vieira Peroni, Amanda Farias Gomes, Alfonso Sánchez-Ayala, Francisco Haiter-Neto, Daniele Manfredini, Célia Marisa Rizzatti-Barbosa

**Affiliations:** 1 Department of Prosthodontics and Periodontology, Piracicaba Dental School, University of Campinas, Sao Paulo 13414-903, Brazil; natalialvarezodont@gmail.com (N.A.-P.); victor_9874@hotmail.com (V.R.M.M.-L.); rizzatti@unicamp.br (C.M.R.-B.); 2 Department of Oral Diagnosis, Piracicaba Dental School, University of Campinas, Sao Paulo 13414-903, Brazil; leo_peroni@hotmail.com (L.V.P.); aamandafg@outlook.com (A.F.G.); haiter64@hotmail.com (F.H.-N.); 3 Department of Dentistry, State University of Ponta Grossa, Paraná 84030-900, Brazil; snzcd@uepg.br; 4 Department of Dentistry, University of Siena, 53100 Siena, Italy; daniele.manfredini75@gmail.com

**Keywords:** temporomandibular disorders, myofascial pain, botulinum toxin type A, bone loss, chronic pain

## Abstract

This study assessed the safety and efficacy of three different doses of BoNT-A for persistent myofascial pain (MFP). One hundred female subjects were randomly assigned into five groups (*n* = 20): oral appliance (OA), saline solution (SS) and three BoNT-A groups with different doses. Pain intensity and pressure pain threshold were evaluated up to 24 weeks after treatment. Adverse effects related to muscle contraction, masticatory performance, muscle thickness and mandibular bone volume were also assessed. Changes over time were compared within and between groups. The “nparLD” package and Wilcoxon signed-rank test were used to analyze the data. BoNT-A reduced pain intensity (*p* < 0.0001) and increased pressure pain threshold (*p* < 0.0001) for up to 24 weeks compared to the placebo. No differences were found between BoNT-A and OA at the last follow-up. A transient decline in masticatory performance (*p* < 0.05) and muscle contraction (*p* < 0.0001), and a decrease in muscle thickness (*p* < 0.05) and coronoid and condylar process bone volume (*p* < 0.05) were found as dose-related adverse effects of BoNT-A. Regardless of the dose, BoNT-A was as effective as OA on MFP. Notwithstanding, due to BoNT-A dose-related adverse effects, we suggest the use of low doses of BoNT-A in MFP patients that do not benefit from conservative treatments.

## 1. Introduction

Persistent myofascial pain (MFP) is a common condition in dentistry with a prevalence of around 45% among subjects with temporomandibular disorders (TMD) [1]. This disorder has a complex pathogenesis expressed as a multifactorial etiology with various systemic and local risk factors causing its fluctuating and self-limiting nature [2].

The limited knowledge on the etiopathogenesis of MFP led to the proposal of numerous approaches to treat this condition [3,4]. Conservative, multidisciplinary, and symptomatic modalities such as oral appliances (OAs), pharmacotherapy, and behavioral and physical treatments are commonly used [5,6,7]. The US-FDA approved botulinum toxin type A (BoNT-A) for the treatment of various muscle disorders, based on its ability to inhibit synaptic exocytosis of acetylcholine and disabling of neural transmission [8]. Additionally, experimental studies demonstrated an analgesic effect of BoNT-A, which is independent of the motor effect [8,9,10], leading to new potential indications for pain conditions, including persistent MFP. 

Despite the variety of published data about the management of persistent MFP with BoNT-A [11,12,13,14], shortcomings like the low number of participants and the lack of standardized protocols of application lead to inconsistent results. Moreover, according to the American Academy of Neurology, BoNT-A is classified as a level B treatment (possibly effective) for MFP [15]. In addition, the evidence of a decrease in masticatory performance (MP), muscle thickness, and bone volume by BoNT-A in different clinical trials [16,17] and in-vivo studies [18,19] raised the concern about the existence of possible adverse events/effects (AEs) that may hinder the benefits of the toxin.

Consequently, the development of new evidence-based and well-designed studies assessing the efficacy, possible AEs, and proper doses of BoNT-A, to establish it as a valid treatment for persistent MFP, became necessary. Therefore, we conducted a randomized controlled clinical trial to assess the efficacy and adverse effects of three different doses of BoNT-A compared with OA and placebo in subjects with persistent MFP. We tested the hypothesis that BoNT-A is more effective than saline solution (SS) and oral appliances for the treatment of persistent MFP.

## 2. Results

**Figure 1:**
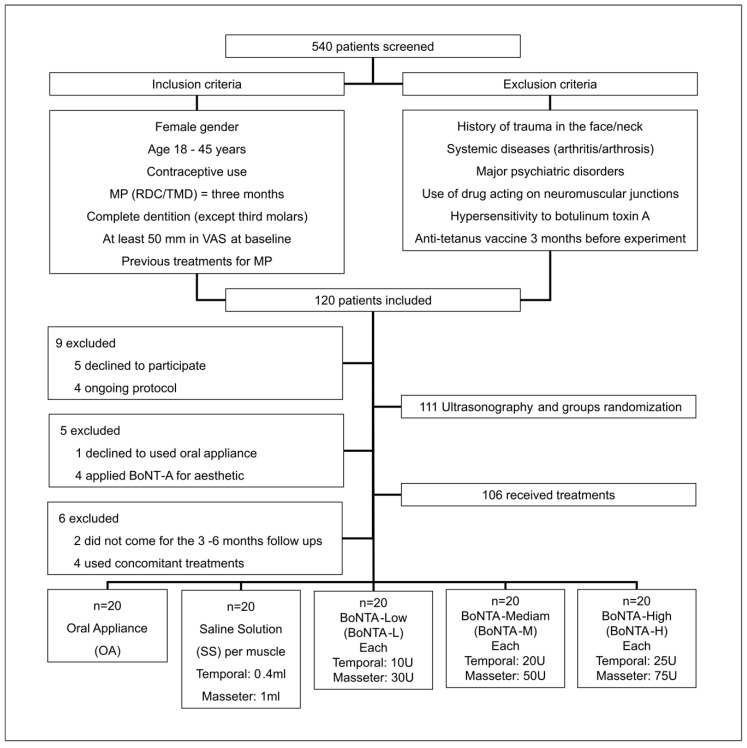
CONSORT diagram for participant enrollment.

One hundred from a total of 540 volunteers were included into the study (mean age = 36.8 ± 5.6) (Figure 1). Characteristics of the included subjects are presented in Table 1.

### 2.1. Subjective Pain (VAS)

**Figure 2:**
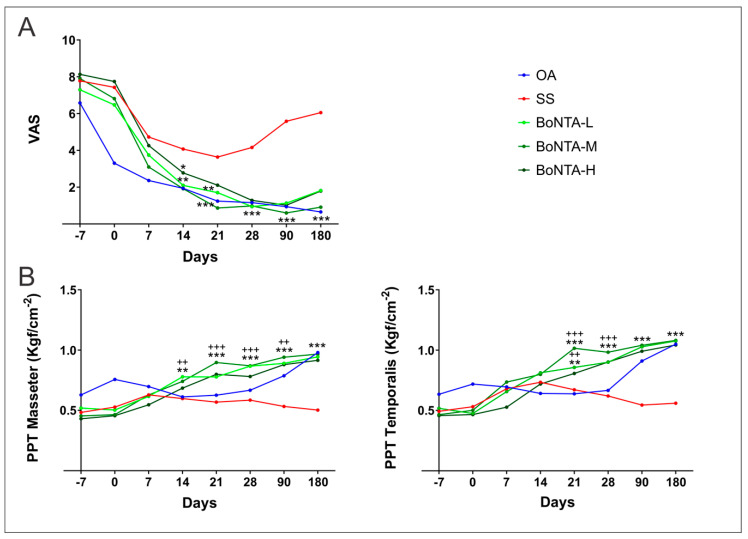
Botulinum toxin effects on subjective pain intensity and in pressure pain threshold versus saline solution and oral appliance over time. OA: oral appliance; SS: saline solution; BoNTA: botulinum toxin type A; L: low; M: medium; H: high. (**A**) Mean; *** = *p* < 0.001 vs. saline solution; ** = *p* < 0.01 vs. saline solution; * *p* < 0.05; (**B**) Mean; *** = *p* < 0.001 vs. saline solution; ** = *p* < 0.01 vs. saline solution; * = *p* < 0.05; Mean; +++ = *p* < 0.001 vs. oral appliance; ++ = *p* < 0.01 vs. oral appliance; group: *p* = 0.023, time: *p* ≤ 0.0001, group x time: *p* ≤ 0.0001.

A significant decrease of subjective pain (Figure 2A) was found in all BoNT-A groups seven days after injections and throughout the experiment (*p* < 0.0001). In addition, no significant differences were found among BoNT-A groups. Compared to the placebo, subjective pain of BoNT-A groups was significantly lower (*p* < 0.0001) after 14 days and up to the end of the study; however, compared with OA, no statistical differences were found after 14 days and up to the end of the study (*p* > 0.05). 

**Figure 3:**
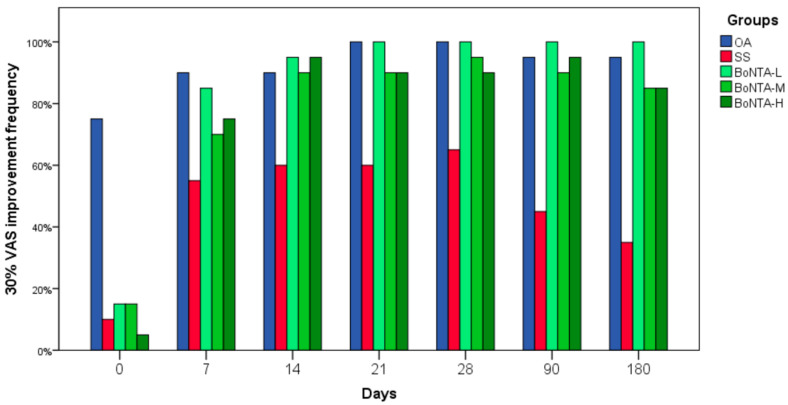
Clinical improvement (30%) in subjective pain.

When individual pain reduction (30%) was calculated in order to know if VAS improvement was clinically significant 20, 19, and 18 patients reported 30% pain reduction in BoNTA-L, M, and H groups respectively, compared to 20 and 13 patients in OA and SS groups respectively at the one month follow up. In the six-month follow up 20, 17, and 17 patients showed a 30% reduction in BoNTA-L, M, and H groups respectively, and 19 and 7 patients in OA and SS groups respectively (Figure 3).

### 2.2. Pressure Pain Threshold (PPT)

The average PPT of the muscles is shown in Figure 2B. In BoNT-A groups, PPT significantly increased (*p* < 0.01) after 14 and 21 days in masseter and anterior temporalis respectively and was maintained until the last evaluation. In addition, BoNT-A significantly improved PPT of both muscles after 21 days compared to placebo (*p* < 0.0001), sustaining this difference up to 180 days. Compared to OA, all BoNT-A groups presented significantly higher values on the 21 (*p* < 0.01) and 28 day (*p* < 0.001) evaluation for the anterior temporalis and in the 14 (*p* < 0.01), 21, 28 (*p* < 0.001) and 90 day (*p* < 0.01) for the masseter. No significant differences were found at the end of the experiment between BoNT-A and OA.

### 2.3. Electromyographic Activity 

**Figure 4:**
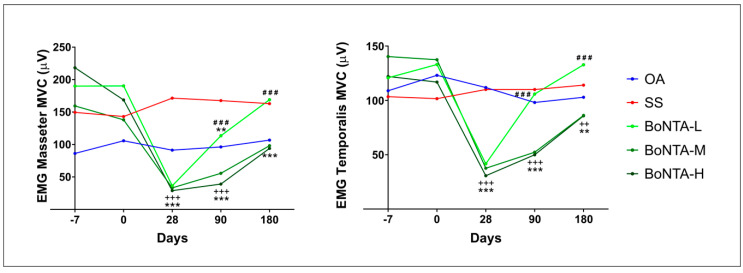
Changes in root mean square scores (RMS µV) of maximum volunteer contraction (MVC) of botulinum toxin compared with saline solution and oral appliance over time. OA: oral appliance; SS: saline solution; BoNTA: botulinum toxin type A; L: low; M: medium; H: high; mean; *** = *p* < 0.001 vs. saline solution; ** = *p* < 0.01 vs. saline solution; +++ = *p* < 0.001 vs. oral appliance; ++ = *p* < 0.01 vs. oral appliance; ### = *p* < 0.001; group: *p* = 0.025, time: *p* ≤ 0.0001, group x time: *p* ≤ 0.0001.

Regarding electromyography (EMG) (Figure 4), BoNT-A significantly decreased muscle activity of masseter and anterior temporalis muscles after 28 days of treatment (*p* < 0.0001) compared with SS and OA. However, just BoNTA-L presented a significant recovery after 90 days and a total recovery of muscle activity at the last follow-up, presenting no significant differences to SS and OA in these evaluations’ points.

### 2.4. Masticatory Performance

BoNT-A significantly decreased MP after 28 days (*p* < 0.0001); however, a significant recovery was found after 90 days and maintained up to 180 days (*p* < 0.05). On the other hand, when BoNT-A and SS groups were compared no significant differences were found just for BoNTA-L in all evaluations (Table 2).

### 2.5. Ultrasound Imaging

Ultrasound imaging (UI) (Table 3) showed a significant decrease in the thickness of all muscles treated with BoNT-A (*p* < 0.0001) in all evaluations. However, when BoNT-A groups were compared to SS, only BoNTA-L showed no significant differences at any evaluation.

### 2.6. Cone Beam Computed Tomography

Concerning cone beam computed tomography (CBCT) (Table 4), the intergroup comparison showed a significant decrease in the coronoid process volume in the BoNTA-M group and the mandible’s head and coronoid process volume in the BoNTA-H group after 90 days of treatment (*p* < 0.05). Comparison between groups showed no differences between BoNT-A and SS groups.

## 3. Discussion

Regardless of the dose, BoNT-A proved to be more effective than SS and at least as effective as OA to treat persistent MFP over 24 weeks. Notwithstanding, dose-dependent AEs were associated with higher doses of the toxin, but not found or easily overcome when a lower dose was employed. These results suggest that lower doses of BoNT-A are more appropriate when this treatment is contemplated for persistent MFP. 

We showed that even lower doses of BoNT-A can reduce subjective pain intensity (VAS) and increase PPT values over 24 weeks, indicating a potential analgesic effect independent of the accompanying dose-dependent motor activity [20]. This analgesic effect was demonstrated by different in vivo and in vitro studies where BoNT-A had peripheral (decreasing levels of substances P, CGRP, and glutamate) [9,21] and even central (axonal transport to sensory regions of trigeminal ganglia) [22,23] action on sensitive neurotransmitters and sensory nerves, or both. Although, decreased muscle contractions due to BoNT-A paralytic effects may also contribute to pain relief [15]. 

In the present study, BoNT-A was significantly better than SS at reducing VAS and increasing PPT values throughout the six months. Likewise, in previous studies [12,13], BoNT-A was able to reduce tenderness to palpation and self-perceived pain compared to the placebo and was maintained over 3 to 4 months. On the other hand, in a well-designed crossover study [11], BoNT-A showed no differences to the placebo even though both treatments achieved a clinically significant reduction of pain (i.e., 30% less pain). In contrast with our study, the small sample size and the decision to treat only one masticatory muscle (masseter) were considered as limitations and may have influenced the obtained results.

OAs are the most used therapy for persistent MFP due to their non-invasive and reversible characteristics [24]. We found no significant differences between BoNT-A and OA to reduce pain over 24 weeks. The improvement of VAS and the increase in the PPT in both therapies showed that BoNT-A is at least as effective as OA. However, AEs derived from OA in muscles were not reported [24] and its effects on bone were not assessed, probably due to its reversible nature. Therefore, the ratio between the effectiveness and the probability of developing AEs should be always be cogitated before considering BoNT-A as an option for persistent MFP.

Within the AEs, a reduction in electrical muscle activity was found after BoNT-A injections for up to the first month. Interestingly, only the BoNTA-L recovered after three months and returned to baseline values after six months. These results support a dose-duration effect for the toxin [25]. In addition, MP which is partially related to muscle activity was reduced after 7 to 28 days of BoNT-A injections, corroborating another study [26] reporting the improvement of MP following BoNT-A applications after 28 days and a completed returned to baseline values after 90 days. Conversely BoNTA-L compared to SS showed no significant differences at any evaluation point. 

Regarding masticatory muscle thickness, a significant decrease in all evaluation periods was also identified among BoNT-A groups. Experimental studies demonstrated that muscle atrophy after BoNT-A injections responded to the decrease of fiber size [27], replacement of contractile tissue for fatty, changes in muscle-fiber composition, or by the increase in mRNA expression of both atrophy (atrogin-1/MAFbx and MURF-1) and muscle regeneration markers (myogenin) [28,29]. Interestingly, reduction in muscle thickness was not significant when BoNTA-L was compared with SS, showing a dose–effect relationship.

The musculoskeletal system maintains a fine regulation between muscles and bones, in which both tissues sustain a cross-talk process that may be affected by the neuroparalytic effect of the toxin. The decrease in the volume of mandible’s structures found in the BoNTA-H group supports this hypothesis. In vivo studies [18,19] have reported bone alterations after 1 to 3 months of a single BoNT-A injection in masticatory muscles. Likewise, clinical trials [16,17] reported changes in mandibular structures after repeated BoNT-A injections. However, we demonstrated that this AE appeared even after a single high-dose injection of BoNT-A (BoNTA-H). Therefore, it could be hypothesized that BoNT-A effects on bone tissues may have a direct toxicity in skeletal cells, increasing the mRNA expression of RANKL, the general inhibition of neurotransmitters could affect other local signaling in bone and cartilage, or even that these effects are consequences of reduced bone loading [18,30].

As far as we know this is the largest study assessing the effectiveness and possible AEs of BoNT-A for persistent MFP. The design of our study, that included standardization of muscle thickness, positive and negative controls groups, three different doses of BoNT-A, and the assessment of its efficacy and safety, allow us to propose a safety margin for this treatment. Following this idea, we consider that in patients that do not get substantial pain relief from conservative treatments, lower doses of BoNT-A are the proper option when this treatment is contemplated for persistent MFP; since the possibility of developing AEs seems to be dose-dependent. Few limitations need to be considered. Longer follow-up periods should be evaluated in order to know the efficacy of a single injection of BoNT-L along time. The included sample was highly selected; therefore, our findings cannot be generalized to a broader population. Finally, even though we demonstrated that BoNT-A produces AEs, its clinical importance remains unclear. Future longitudinal studies should assess if AEs remain for longer periods of time and even if multiple applications of low doses of BoNT-A could develop AEs.

## 4. Conclusions

It can be concluded that BoNT-A is at least as effective as OA for persistent MFP. However, due to dose-related AEs, conservative treatments (e.g., OA) should be the first option for MFP. Notwithstanding, we suggest the usage of low doses of BoNT-A in patients that do not get substantial pain relief from conservative therapies. Further studies must be performed to refine BoNT-A’s indications.

## 5. Methods 

### 5.1. Subjects

The present randomized, controlled clinical trial included 100 consecutive diagnosed subjects with persistent MFP according to the Portuguese version of the Research Diagnostic Criteria for Temporomandibular Disorders [31] at the TMD Clinic of Piracicaba Dental School, University of Campinas, São Paulo, Brazil, by two calibrated researchers not involved in any other processes of the study (kappa coefficient = 0.80). Female subjects were selected according to the criteria shown in Figure 1. The Ethics Committee of Piracicaba Dental School (CAAE # 22953113.8.0000.5418) (Date: 04/02/2014) and the Brazilian Registry of Clinical Trials (ReBEC RBR-2d4vvv) approved the study. All subjects provided a written informed consent before inclusion. 

### 5.2. Randomization

Since BoNT-A effects are muscle-size dependent, subjects were submitted to ultrasonography of the masseter and anterior temporalis muscles to evaluate muscle thickness and equally divide them into above or below the median in each group (5.8 mm/anterior temporalis; 11.6 mm/masseter). Subjects were then randomly assigned into one of the following treatment groups (*n* = 20): oral appliance (OA; positive control group), saline solution (SS; negative control group), BoNT-A low (BoNTA-L), BoNT-A medium (BoNTA-M), and BoNT-A high (BoNTA-H) (Figure 1). For this allocation, computer software (Isfahan, Iran https://random-allocation-software.software.informer.com/2.0/) was operated by a technician not involved in any other procedures in the study. Subjects and investigators were masked to BoNT-A and SS assignments, while investigators assessing the outcomes were masked to all treatment assignments.

### 5.3. Treatments

#### 5.3.1. Counseling (CLS)

CLS consisted of giving information about the anatomy and physiology of the stomatognathic system, the etiology and possible prognosis of persistent MFP, and given self-care strategies to control parafunctions. The same trained clinician performed CSL at the first appointment. 

#### 5.3.2. Oral Appliance (OA)

The OA was a flat intraoral device covering all superior teeth, made of transparent thermo-polymerized acrylic resin. Subjects were instructed to use the OA during sleep throughout the study (Figure 2). Adjustments (a slight canine and anterior guidance) were performed when required.

#### 5.3.3. BoNT-A and SS

BoNT-A (100 U; Botox, Allergan, Irvine, California, CA, USA) was reconstituted using non-preserved sterile saline solution 0.9%. To blind the applications of BoNT-A and SS, each muscle received an injection containing 1 mL of the correspondent diluted BoNTA-L/M/H doses or SS by an investigator who was not involved in the dilution process. Doses of BoNT-A were based on previous reports [11,12,13,14] and assigned according to group distribution (Figure 1). Bilateral intramuscular injections were performed using a 1-mL syringe with a 30-gauge needle. Subjects were asked to clench to delimit the muscle area (masseter and anterior temporalis) and a total of 5 injections per muscle with a separation of 5 mm were applied. 

### 5.4. Outcomes

**Figure 5:**
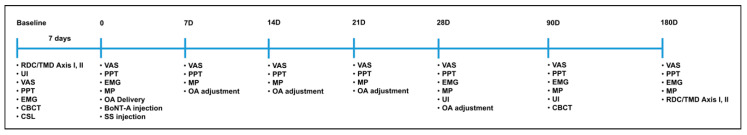
Study flow diagram. RDC/TMD: Research Diagnostic Criteria for Temporomandibular Disorders; VAS: visual analogue scale; PPT: pressure pain threshold; EMG: electromyography; MP: masticatory performance; UI: ultrasound imaging; CBCT: cone beam computed tomography; CSL: counseling; OA: oral appliance; BoNT-A; botulinum toxin type A; SS: saline solution D: day.

Subjects were evaluated eight times throughout the 6 months of study. Outcomes at each evaluation interval are presented in Figure 5. 

#### 5.4.1. Primary Outcomes

##### Subjective Pain: Visual Analog Scale (VAS)

The VAS is a 100 mm horizontal line anchored by word descriptors at each end. The left end is labeled “no pain,” and the right “worst pain imaginable” [32]. Participants were instructed to mark a point on the line representing the level of current, worst, and average pain. Mean values were used for statistical analyses.

##### Pressure Pain Threshold (PPT)

The PPT was assessed bilaterally for masseter and anterior temporalis muscles (most prominent part in functional test) in a relaxed position using a digital algometer 1 cm^2^ circular point (Kratos DDK-20. São Paulo, Brazil) used to exert pressure on the muscles. An increasing and constant pressure of 0.5 kg/cm^2^ was applied perpendicular to the surface of the skin [33]. Subjects were instructed to indicate when the pressure became painful. This procedure was executed by a single calibrated operator (Kappa = 0.89). PPT measurement sites were aligned with BoNT-A injections sites. 

#### 5.4.2. Secondary Outcomes

##### Electromyography (EMG)

To record the electromyographic signal, ADS 1200 equipment (Lynx Electronic Technology Ltd., Sao Paulo, Brazil) was used by a single calibrated operator (Kappa = 0.80). Bipolar electrodes were fixed in the most prominent part of each muscle after skin preparation and the reference electrode placed on the manubrium of the sternum of the volunteers [34]. The Lynx AqDa- dos 7.02 and Lynx AqD Analysis 7.0 (Lynx Electronic Technology Ltd., Sao Paulo, Brazil) software were used to acquire simultaneous signals and to process the root mean square values (RMS; expressed in µV). Electrical activity of the muscles was recorded in maximum voluntary contraction. For this, a parafilm M (American National Can, Chicago, IL, USA) was placed bilaterally on the molars and subjects were instructed to clench with maximum force and maintain for 5 s. This step was repeated three times. To avoid fatigue, a 2-min period of rest between collections was allowed [35]. Mean values were used for statistical analyses.

##### Masticatory Performance (MP)

The multiple sieve method was used to assess MP. Cubical test foods of 5.6 mm were molded with condensation silicone (Optosil Comfort; Heraeus Kulzer, Hanau, Germany) using a stainless-steel matrix. Then, each subject was asked to chew 17 cubes (3.4 g) 20 times in a habitual manner [36]. 

Afterward, subjects spat out the comminuted particles onto filter paper. After draining and disinfection, the filter with the particles was dried in an oven at 80 °C for 25 min. Each sample was sieved through a stack of up to 10 sieves with aperture sizes of 0.50 to 5.60 mm based on a √^2^ progression in a shaker (Bertel Indústria Metalúrgica Ltd.a, Caieiras, Brazil) for 20 min. The particles retained in each sieve were weighed on a 0.001 g balance (Mark 2060; Bel Engineering, Lombardy, Italy). The masticatory performance was calculated using a nonlinear regression (Rosin Rammler equation) [36].

##### Ultrasound Imaging (UI)

Thickness of both masseters and anterior temporalis muscles during maximum volunteer contraction (MVC) was calculated using real-time UI (SSA-780 A-APLIO Mx, 38 mm/7-18 MHz; Toshiba Medical System Co., Tokyo, Japan) by a single calibrated operator (Kappa = 0.86). Muscle thickness was measured directly on the instrument’s screen with an accuracy of 0.01 mm [37]. 

##### Cone Beam Computed Tomography (CBCT)

The volume of the coronoid and condylar process of the mandible was assessed through CBCT (Picasso Trio 3D, 85 kVp, 5 mA, voxel of 0.2 mm and a 12 × 8.5 cm FOV; Vatech, Hwaseong, South Korea). For this, CBCT multiplanar reconstructions were assessed with the semi-automatic mode of ITK-SNAP 3.4.0 segmentation software (Cognitica, Philadelphia, PA, USA) [38]. After segmentation, bone volumes were measured in cubic millimeters. The evaluations were performed by two oral radiologists (ICC > 0.95).

Due to limited funding sources we decided to assess MP, UI, and CBCT just in BoNT-A groups and in SS group.

### 5.5. Statistical Analysis

The sample size was based on the average pain scores of a previous study [12]. Power calculation showed that 8 patients per group would demonstrate more than 90% power when α = 0.05. Variables were explored and analyzed descriptively with mean and standard deviations of the quantitative measures for groups and periods. Variables were tested for adherence to normal distribution using the Shapiro–Wilk test and differences between the variances by the Levene test. There was no normal distribution and there was a significant difference between variances (*p* < 0.001). Then, a two-way non-parametric test alternative was performed to analyze the interaction of time-points and treatment groups as simultaneous influence factors on variables. For non-parametric multiple comparison, in pairs, Wilcoxon signed-rank test with Bonferroni correction was used to show interaction between the time-points (paired measurements) (α = 0.05), and Mann–Whitney U with Bonferroni correction to groups (independent measurements) (α = 0.05) [39]. All variables were considered numerical, for VAS, a binomial analysis also was applied to consider responders with 30% improvement (clinically significant). The software used for the statistical analysis was the R version 3.5.2 (R Foundation for Statistical Computing, Viena, Austria).

## Figures and Tables

**Table 1:** Characteristics of the included subjects.

Characteristics	*n*
Mean age	36.8 ± 5.6
Gender: Female	100
Education	
Elementary school	12
High school	39
University	49
Occupation	
Student	24
Employed	62
Unemployed	14
Pain duration	
6 months	41
1–2 years	37
3 or more	22
RDC/TMD diagnoses	
Myofascial pain	53
Myofascial pain/Arthralgia	12
Myofascial pain/Disc displacement with reduction	27
Myofascial pain/Disc displacement without reduction	8

**Table 2:** Masticatory performance values (mean and standard deviation) between botulinum toxin and saline solution groups.

Groups	Periods of Evaluation						
	0 D	7 D	14 D	21 D	28 D	90 D	180 D
SS	6.17 ± 0.66 Ba	5.84 ± 0.74 Ca	5.76 ± 0.78 Ba	5.67 ± 0.87 Ca	5.69 ± 0.86 Ba	5.09 ± 1.07 Cb	4.97 ± 1.10 Bb
BoNTA-L	5.50 ± 0.89 Aab	6.14 ± 0.71 CBc	6.09 ± 0.64 Bc	6.13 ± 0.76 CBc	6.20 ± 0.44 Ac	5.53 ± 0.70 BCb	5.07 ± 0.80 Ba
BoNTA-M	5.93 ± 0.88 ABa	6.84 ± 0.50 Abc	6.94 ± 0.59 Ac	6.77 ± 0.70 Abc	6.65 ± 0.61Ac	6.28 ± 0.79 BCb	6.19 ± 0.74 Aa
BoNTA-H	5.94 ± 0.83 ABa	6.42 ± 0.63 ABbc	6.62 ± 0.49 Ab	6.65 ± 0.58 ABb	6.43 ± 0.66 Ab	6.07 ± 0.72 ABac	5.85 ± 0.88 Aa

Uppercase letters in vertical represent significant differences among groups *(p < 0.05)*; lowercase letters in horizontal denote significant differences among assessment time points within groups (*p* < 0.05); BoNT-A: botulinum toxin type A; SS: saline solution; D: days; group: *p* = 0.0007; time: *p* ≤ 0.0001; group x time: *p* = 0.0014.

**Table 3:** Mean (standard deviation) values of muscle thickness during maximum voluntary contraction (MVC) of botulinum toxin versus saline solution groups.

Muscle/Groups	Periods of Evaluation		
	0 D	30 D	90 D
Temporalis in MVC			
Saline solution	2.45 ± 1.05 Aa	2.45 ± 1.27 Aa	2.57 ± 1.51 Aa
BoNTA-Low	2.32 ± 0.71 Aa	1.70 ± 0.65 ABb	1.70 ± 0.59 ABb
BoNTA-Medium	2.13 ± 0.88 Aa	1.40 ± 0.55 Bb	1.47 ± 0.62 Bb
BoNTA-High	1.99 ± 0.82 Aa	1.54 ± 0.71 Ba	1.41 ± 0.43 Ba
Masseter in MVC			
Saline solution	11.70 ± 1.88 Aa	12.04 ± 2.11 Aa	12.09 ± 1.79 Aa
BoNTA-Low	12.37 ± 1.75 Aa	11.75 ± 1.47 ABb	11.6 ± 1.70 ABb
BoNTA-Medium	11.90 ± 1.55 Aa	9.88 ± 1.83 Bb	10.49 ± 1.72 Bb
BoNTA-High	12.34 ± 1.68 Aa	10.44 ± 1.26 Bb	11.38 ± 1.67 Bb

Uppercase letters in vertical represent significant differences among groups *(p* < 0.05); lowercase letters in horizontal denote significant differences among assessment time points within groups *(p* < 0.05); MVC: maximum volunteer contraction; BoNT-A: botulinum toxin type A; D: days; group: *p* ≤ 0.0001; time: *p* ≤ 0.0001; group x time: *p* ≤ 0.0001.

**Table 4:** Changes in mandible’s head and coronoid process bone volume of botulinum toxin against saline solution groups.

Mandible Structures/Groups	Periods of Evaluation	
	0 D	90 D
Mandible head		
Saline solution	1249.91 ± 377.28 Aa	1257.23 ± 396.93 Aa
BoNTA-Low	1495,79 ± 491,02 Aa	1508,54 ± 478,23 Aa
BoNTA-Medium	1573,31 ± 418,47 Aa	1580,88 ± 407,53 Aa
BoNTA-High	1516,46 ± 429,90 Aa	1483,69 ± 405,61 Bb
Coronoid Process		
Saline solution	217.2 ± 77.28 Aa	210.9 ± 50.6 Aa
BoNTA-Low	213.6 ± 122.3 Aa	203.1 ± 213.8 Aa
BoNTA-Medium	195.6 ± 118.1 Aa	164.3 ± 135.9 Bb
BoNTA-High	207.9 ± 108.7 Aa	189.2 ± 93.8 Bb

Uppercase letters in vertical represent significant differences among groups *(p* < 0.05); lowercase letters in horizontal denote significant differences among assessment time points within groups *(p* < 0.05); BoNT-A: botulinum toxin type A; D: days; group: *p* = 0.0056; time: *p* = 0.032; group x time: *p* = 0.002.
